# Supplementary figures and images for: Educational patterns of health behaviors and body mass index: A longitudinal multiple correspondence analysis of a middle-aged general population, 2007–2016
Source: PLoS One. 2023 Dec 1;18(12):e0295302. doi: 10.1371/journal.pone.0295302 (PMC10691680; doi:10.1371/journal.pone.0295302)

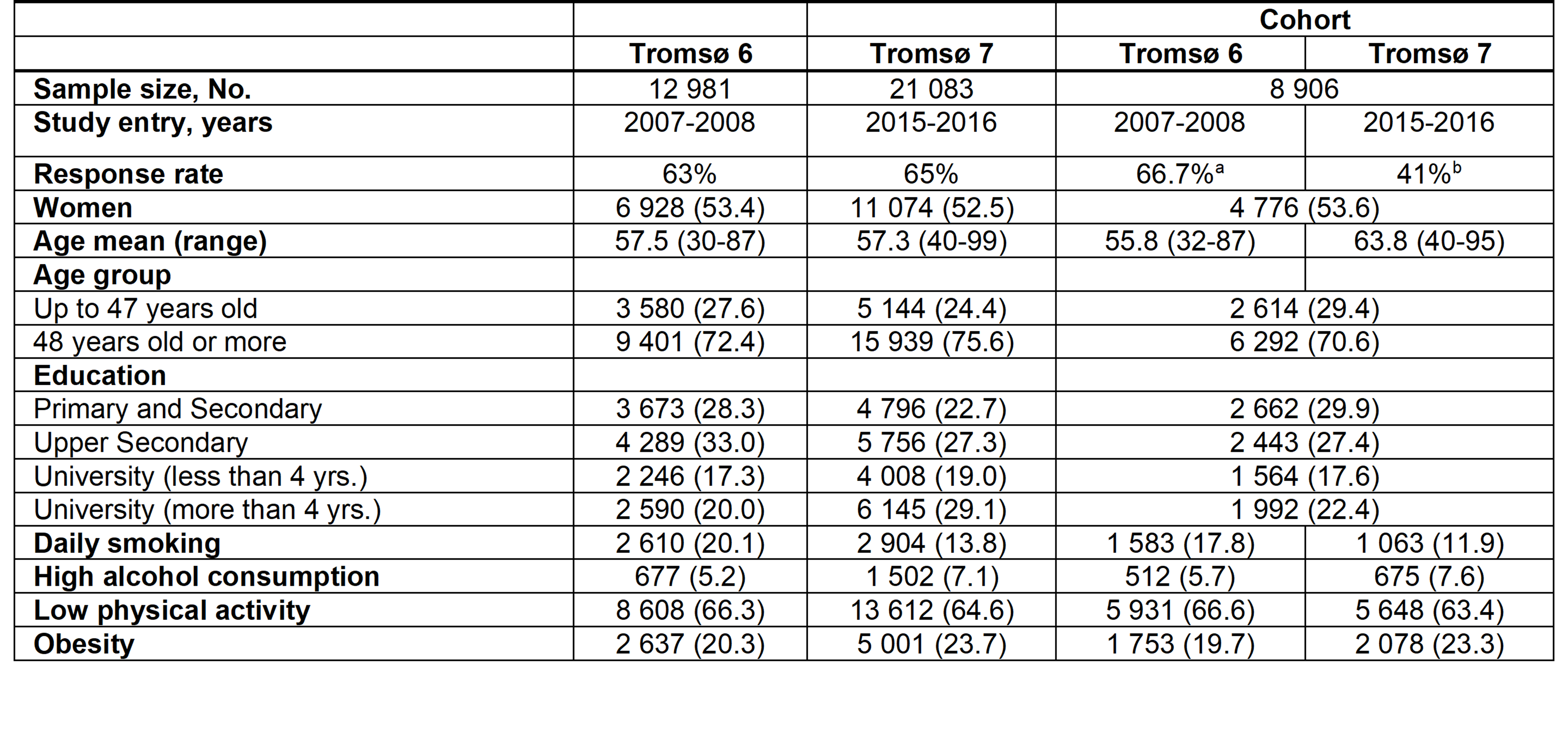

Supplement: S1 Table — a Percentage of participants in Tromsø 6 that also participated in Tromsø 7. b Percentage of participants in Tromsø 7 that also participated in Tromsø 6. High alcohol consumption: more than 14 units per week for men and 7 units per week for women. Low physical activity: Less than 150 minutes per week. Obesity: body mass index of 30 kg/m2 or more. (TIF) [file pone.0295302.s001.tif]
