# Supplementary material for: Educational patterns of health behaviors and body mass index: A longitudinal multiple correspondence analysis of a middle-aged general population, 2007–2016
Source: PLoS One. 2023 Dec 1;18(12):e0295302. doi: 10.1371/journal.pone.0295302 (PMC10691680; doi:10.1371/journal.pone.0295302)
Supplement: Attachment — Submitted filename: Patterns of health behavior and socioeconomic status - review.docx [file pone.0295302.s002.docx]

**Overall comment:s**

The entire manuscript needs proofreading—I noticed several spelling/grammar mistakes, and the writing could be improved. Certain sections need to be expanded (e.g. interpretation of results, exploration of mechanisms), while some could be shortened (e.g. how MCA works).

The figures/tables need to be revised, they are poorly organized, which makes them hard to follow For example, why was Table 1 stratified by gender, when gender was not discussed at all in the manuscript? And why was Table 2 never referenced? Is column percentage really the best way to present data?

It is unclear (at least based on the manuscript) if the changes in behaviors are measured at individual level or group level. And additional analyses might

**Introduction**

“Socioeconomic differences in health persist and are growing markedly, even in countries with good **social circumstances** (1-3).”

Please explain what you meant by social circumstances

“Unhealthy behaviors, such as smoking, harmful alcohol consumption, physical inactivity, poor diet and **high BMI** are risk factors for chronic diseases (7, 8)”

High BMI is not a behavior; and it is more like an intermediate or measure of outcome in your case

“Monitoring social inequalities in the burden of chronic diseases and **its** determinants can facilitate the development of policies to improve health equality”

Please proofread the entire manuscript

**Materials and methods**

“The Tromsø Study is a **prospective cohort** of the residents of the municipality of Tromsø”

Just to confirm, these are the same people followed over time? I think you mentioned it later but you need to clarify it here too. Is it an open cohort? What are the retention rate? Etc.

“**Level of education** was used as indicator of socioeconomic status due to previous research showing education as one of the main indicators of socioeconomic status in the Nordic countries (35)”

I would like to see more justification of using only education as indicator, since socioeconomic status is fairly complex and could be measured in many ways. You might want to compare several measures since it is the key exposure/factor in your study

**Results**

“Only 6.3% of the respondents reported a **change in alcohol consumption** behavior, followed by 13.1% in daily smoking.”

Please only describe things you meant to interpret—here you combined both health and unhealthy changes—which might be pointless to bring up

“In **dimension 1**, BMI and BMI change are indicated to have the largest discriminatory power, with “remain normal weight” on one side, and three different unhealthy BMI categories on the right side (“remain obese”, “overweight to obese” and “normal to overweight”).”

There was a very long section on MCA in the methods section, yet here there was no explanation on what the dimensions mean. Need to expand this section for those who aren’t familiar with MCA.

“Level of education was included as a supplementary variable. Supplementary points define additional profiles that are not used to establish the solution space but are projected onto the space afterwards.”

Does that mean this study is purely descriptive? You might want to consider additional analyses like regression where you could account for confounding. Also, since there’s no measure of association, you need to be cautious when discussing biases in later section.

**Discussion**

“A longer exposure time could further explain the **mechanism** of *differential susceptibility* (47)”

The mechanism was barely explored in the manuscript
