# Supplementary material for: Educational patterns of health behaviors and body mass index: A longitudinal multiple correspondence analysis of a middle-aged general population, 2007–2016
Source: PLoS One. 2023 Dec 1;18(12):e0295302. doi: 10.1371/journal.pone.0295302 (PMC10691680; doi:10.1371/journal.pone.0295302)
Supplement: Attachment — Submitted filename: Response to Reviewers.docx [file pone.0295302.s003.docx]

**Reviewer #1:** Overall comments
1. The entire manuscript needs proofreading—I noticed several spelling/grammar mistakes, and the writing could be improved. Certain sections need to be expanded (e.g. interpretation of results, exploration of mechanisms), while some could be shortened (e.g. how MCA works).

Thanks for this comment. We have now revised the language and proofread the manuscript. We have expanded our interpretation of results, mechanisms and shortened the section on how Multiple Correspondence Analysis works.

2. The figures/tables need to be revised, they are poorly organized, which makes them hard to follow For example, why was Table 1 stratified by gender, when gender was not discussed at all in the manuscript? And why was Table 2 never referenced? Is column percentage really the best way to present data?

Figures and tables have been revised according to the organization of the revised manuscript, assured they were referenced in the main body of the text. In addition, we have added an additional analysis that explore sex differences.

3. It is unclear (at least based on the manuscript) if the changes in behaviors are measured at individual level or group level. And additional analyses might

Thanks for making us aware of this! We have now elaborated on this in our methods section, lines [159-161] , making it clearer that changes are measured at the individual level.

4. Introduction
“Socioeconomic differences in health persist and are growing markedly, even in countries with good social circumstances (1-3).”
Please explain what you meant by social circumstances.

We have reworded this sentence to explain what we meant by social circumstances. See line no. [45-46].

5. “Unhealthy behaviors, such as smoking, harmful alcohol consumption, physical inactivity, poor diet and high BMI are risk factors for chronic diseases (7, 8)”
High BMI is not a behavior; and it is more like an intermediate or measure of outcome in your case

Thanks for pointing this out. We have changed this sentence to make this distinction clearer throughout the manuscript.

6. “Monitoring social inequalities in the burden of chronic diseases and its determinants can facilitate the development of policies to improve health equality”
Please proofread the entire manuscript.

Thanks for this comment. We have now revised the language and proofread the manuscript.

7. Materials and methods
“The Tromsø Study is a prospective cohort of the residents of the municipality of Tromsø”
Just to confirm, these are the same people followed over time? I think you mentioned it later but you need to clarify it here too. Is it an open cohort? What are the retention rate? Etc.

We are very grateful for this comment. More detail about the Tromsø study has been added to clarify that we are following the same people over time, and the retention rate can be seen in the table of descriptive characteristics.

8.“Level of education was used as indicator of socioeconomic status due to previous research showing education as one of the main indicators of socioeconomic status in the Nordic countries (35)”
I would like to see more justification of using only education as indicator, since socioeconomic status is fairly complex and could be measured in many ways. You might want to compare several measures since it is the key exposure/factor in your study.

Good points! In terms of education as an indicator of SEP, we have now added information to justify it as indicator of socioeconomic status, lines [136-144]. For example, education is a stable measure that is maintained even if respondents change their employment status over time, which is ideal for the design of our study. An additional advantage is that the education variable in the lates waves of the Tromsø Study has been recently validated. Since the variable of income remains self-reported to this date, we could not add a suitable comparison between several measures of SEP.

9. Results
“Only 6.3% of the respondents reported a change in alcohol consumption behavior, followed by 13.1% in daily smoking.”
Please only describe things you meant to interpret—here you combined both health and unhealthy changes—which might be pointless to bring up

Good point! We have now distinguished between the behavior factors that underwent more change versus those that changed less. Interestingly, smoking and alcohol consumption were the questions in which a larger number of respondents in the cohort sample reported the same answer in 2007/08 and in 2015/16. In contrast, a larger number of respondents reported a different answer or fell into a different category with respect to BMI and physical activity at the follow-up. We consider this an important observation and therefore discuss this further on lines [176-184].

10. “In dimension 1, BMI and BMI change are indicated to have the largest discriminatory power, with “remain normal weight” on one side, and three different unhealthy BMI categories on the right side (“remain obese”, “overweight to obese” and “normal to overweight”).”
There was a very long section on MCA in the methods section, yet here there was no explanation on what the dimensions mean. Need to expand this section for those who aren’t familiar with MCA.

Thanks for making us aware of this. We have now reworded our results section by removing the overtechnical use of language used in the field of MCA and have now kept only the information most relevant to the main findings for our results’ interpretation.

11. “Level of education was included as a supplementary variable. Supplementary points define additional profiles that are not used to establish the solution space but are projected onto the space afterwards.”
Does that mean this study is purely descriptive? You might want to consider additional analyses like regression where you could account for confounding. Also, since there’s no measure of association, you need to be cautious when discussing biases in later section.

Thank you for pointing out this about confounding! We could account now for some of the confounding in the complex relationship between education and health behavior by stratifying the analysis by age and gender. Although we don’t perform any specific hypothesis testing in the manuscript, our study is not deemed as purely descriptive, see line [152-157]. MCA is a visualization of the measure of associations between a set of variables. We will look further into other statistical ways of analyzing this relationship in future work, as also noted in the discussion.

12. Discussion
“A longer exposure time could further explain the mechanism of differential susceptibility (47)”
The mechanism was barely explored in the manuscript.

We have expanded our discussion section with regard to this concept.

**Reviewer #2:**

13.This is an interesting manuscript with a novel approach to measuring the mediators of health inequalities through the life course.
The authors’ rationale is clear, as are the results here-presented. However, I believe the manuscript has some limitations that need to be revised and some gaps that need to be filled in.

Introduction:
When mentioning health differences, in line 56, please specify which differences (all-cause mortality) as the size of inequalities could vary according to the health indicator. Also, mention how these differences are measured: rate ratios vs rate differences.

Thanks for pointing this out. We have now included more information about the European comparison by Mackenback and colleagues.

14. In line 63 the authors state that little is known regarding inequalities in multiple health behaviours. The authors must consult Lakshman R, et al. article (Lakshman R, et al. Association between area-level socioeconomic deprivation and a cluster of behavioural risk factors: cross-sectional, population-based study. Journal of Public Health. 2010 Sep 29;33(2):234-45)

Thanks for making us aware of this important work by Lakshman and colleagues! We agree that *little* is not the appropriate term, therefore in line 63 we originally wrote *less is known.* There is less research with longitudinal design about socioeconomic inequalities in multiple health behaviors compared to the number of cross-sectional studies. We could not fit this work by Lakshman and colleagues because we wanted to focus on highlighting the studies with longitudinal or repeated cross-sectional design and/or from Scandinavian populations.

15. In lines 69 to 72 the authors seem to oversimplify (and incorrectly report) the reference:
[2003] “When compared to the Healthy group, individuals in the Apathetic group were younger (odds ratio [OR] = 0.92), male (OR = 2.89), lower income (OR = 0.87), less educated (OR = 0.74), more likely to be Black (OR = 1.30), and less likely to be Other (OR = 0.79) versus White. The Binge-drinking group was younger (OR = 0.72), male (OR = 7.38), lower income (OR = 0.85), less educated (OR = 0.57), and less likely to be Hispanic (OR = 0.76) versus White than the Healthy group.
[2015] “Participants in the Apathetic group were more likely to be younger (OR = 0.87), male (OR = 1.41), lower income (OR = 0.63), less educated (OR = 0.45), Black versus White (OR = 1.86), and White versus Hispanic (OR = 0.67) than the Physically Active group. Participants in the Binge-drinking group were younger (OR = 0.73), male (OR = 3.28), lower education (OR = 0.53), lower income (OR = 0.79), Black versus White (OR = 1.31), White versus Hispanic (OR = 0.70), and Other (OR = 0.66) when compared to the Physically Active group.”

Thank you for expanding this for us. We have now expanded the findings from this research article and corrected our reporting of these results.

16. Further, the authors must confront these findings from the US to those from other authors and studies, as from Cutler et al. (Cutler DM, Lleras-Muney A. Understanding differences in health behaviors by education. Journal of health economics. 2010;29(1):1-28)

Thanks for making us aware of this interesting study by Cutler and Lleras-Muney. We have now discussed our findings related to this and other work.

17. Considering lines 84-85 (“observations from longitudinal studies have suggested that a large percentage of individuals follow a pattern of long-term adherence to the same health behaviors”) the authors must clearly state (1) what they expect this study to add to the literature and (2) their research hypothesis.

We have now clearly stated what we expect our study will add to the current literature in line no. [63-67] and [86-92].

18. Methods:
The use of BMI as a proxy for healthy eating seems inappropriate, as BMI importantly depends not only on the diet but also on the intensity and frequency of physical activity, among others. It seems more adequate to remove this variable from the study or assume it as BMI (and not a proxy).

Thanks for this insight! We have now made sure BMI is not assumed as a proxy for healthy eating and now is assumed it as BMI.

19. It is unclear if the authors grouped the categories of the variables of interest or if they used the original categorization. The authors must explain the rationale behind the decision of aggregation/no aggregation of these categories (for example, is low BMI considered normal weight?).

Thanks for pointing out that there was some justification missing in our methods section. We have now added information about the way categories were grouped in the methods section.

20. The authors must state if (and how) sex and age were used in the multiple correspondence analysis.

We have now stated how age and sex were used in the analyses.

21. Results:

1) The authors must clarify what means, for the study aim, (1) the inertia of the two dimensions, (2) BMI’s “largest discriminatory power”, (3) the contributions of physical activity and alcohol consumption to the spread of dimension 1, (4) the considerable discriminatory power of BMI and smoking on dimension 2.

We have now reworded our results section to remove the overtechnical use of language and keeping only the information most relevant to the main findings for our results’ interpretation.

22. A legend is needed in Figure 1.

We have revised our figures including their legends.

23. Discussion:

1) Besides the ‘differential susceptibility’ other factors and pathways may explain (1) the higher risk of low-educated individuals to have unhealthy behaviours and (2) their difficulty in changing them, besides the (3) overall population to change behaviours. Material and immaterial resources (see Mackenbah et al. The persistence of health inequalities in modern welfare states: The explanation of a paradox. SSM 2012), psychosocial stress and available coping mechanisms, environmental opportunities, the capacity of the health system to support people and their knowledge on how to navigate it, social norms and social control, can be some of the hypotheses that should be enunciated in this section.

Thanks for pointing us in the direction of this important work. We have now mentioned the literature behind the diverse pathways in our discussion section.

24. The reader probably will know little about the Norwegian context: it would be important that the authors contextualize some of the reasons behind the persistence of unhealthy behaviors in the life course, especially among the least educated.

Thanks for pointing us in the direction of this important work. Literature about the mechanisms behind the relationship between education and health behavior in the Norwegian context has been added to our discussion.

25. The authors mention a selection bias regarding education - couldn’t there be a bias regarding behaviours, ie., couldn’t people with a higher number of unhealthy behaviours refrain from participating in subsequent waves?

Excellent point, we have mentioned this on lines [312-317].

26. How may these selection biases impact this study's results?

We have now included more about the potential impact of the selection bias.

27. As stated above, BMI should not be used as an indicator of healthy eating. It strongly depends on physical activity (besides other factors).

Thanks for this insight! We have now made sure BMI is not assumed as a proxy for healthy eating and now is assumed it as BMI.

**Reviewer #3:**

28. This is a two wave survey (longitudinal) on 4-5 health behaviors and one SES indicator being educational attainment. While past predicts future, healthy behaviors tend to covary, and they are associated with higher educational attainment. These are partially known, but that does not make me less interested in the results if:
1- The paper discusses that overlap between SES and various health behaviors suggest there might be a sub-additive effects of various health behaviors, because of overlapping mechanism. That means, the total effect is probably smaller than sum of the effects, because health behaviors tend to manifest in the same individual. This subadditive versus multiplicative/synergistic effect if of interest of the literature.

Thanks for this important insight! We have now welcomed the literature about this point in our discussion section.

29. 2- The paper needs to go beyond main effects that assume all effects are universal across subgroups. We did not see the distribution of immigrants and native individuals and ethnic groups. A large body of literature shown that the effects of education is maximum in native people and minimum in marginalized people such as immigrants because the system does not similarly value their education, and their education does not become income etc whoch is needed for healty diet and exercise. so, these diminished returns of education based on marginalization status should be tested. If all associations are universal (no interaction), then your country is a very non-discriminatory context, but if marginalized people with high education still engage in health risk behaviors, it is probably because they work in worse jobs and have lower income nad higher stress. These to be tested and discussed based on a very well-established litertaure on MDRs (diminished returns). Nothing should be assumed to be universal. One size does not fit all.

After these comments are addressed, I can review the paper again and suggest publication.

We completely agree that this is a very important issue to take into account. We have now mentioned this aspect in the discussion section .
