# Supplementary material for: Educational patterns of health behaviors and body mass index: A longitudinal multiple correspondence analysis of a middle-aged general population, 2007–2016
Source: PLoS One. 2023 Dec 1;18(12):e0295302. doi: 10.1371/journal.pone.0295302 (PMC10691680; doi:10.1371/journal.pone.0295302)
Supplement: Attachment — Submitted filename: Response to Reviewers 2.docx [file pone.0295302.s004.docx]

Strengths

• This was an interesting study that longitudinally assessed multiple health behaviors in the same study sample over time that included both men and women.

• The longitudinal design, large sample size, and decision to assess multiple health behaviours were strengths.

• MCA analysis was novel.

**Thank you for acknowledging the strengths of our research study.**

Weaknesses

• Introduction page 12, line 48: high BMI is not a behaviour as the others are, but is a clinical risk factor that is the result of poor diet and low physical activity - if you mention BMI here you should also mention high blood pressure, cholesterol etc. Same for line 56. If the focus of the paper is to highlight socioeconomic differences in health behaviours, BMI should not be included in this list.

**Thank you for your comment. As you point out, it is important that BMI is not referred as a behavior. We have now made the necessary changes throughout the paper to make it clearer that BMI is not referred as a behavior.**

• Related to above, the authors continue to refer to BMI as a poor health behaviour, which it is not. It is the result of poor health behaviours (poor diet and low levels of physical activity) – and in some cases, BMI does not reflect either of these (e.g., in athletes with high muscle mass relative to height). Further, BMI (weight) may increase over time not due to poor health behaviours, but medication side effects and hormonal changes associated with pregnancy or menopause (in women). This is another reason why it is conceptually and methodologically inappropriate to define BMI as a health behaviour, so any reference to BMI being a health behaviour should be deleted and/or edited throughout the manuscript. The previous reviewers had also made this comment but this has not been addressed by the authors.

**Thank you for your comment. We have now deleted any reference to BMI as a behavior throughout the manuscript. For instance, the title is now changed to: "Educational patterns of health behavior *and* BMI…” to differentiate between BMI and the other variables related to health behavior.**

• The main outcome measures (health behaviours) were not very sensitive. For example, smoking was assessed as current, past or never – but did not include a measure of volume or duration, which is critical for estimating impacts on health. Pack years would have been a more sensitive measure.

**We understand the objection. While the question to assess smoking behavior was not very sensitive, current health guidelines do not establish any safe threshold for smoking behavior regarding both volume of consumption and frequency. We have made sure to include these aspects in our discussion section (see line 355-359).**

Also, participants were defined as physically active only if they reached the 150min/week threshold, but this obscures the ability to observe a dose-response relationship between education and physical activity. Further, the authors calculated physical activity minutes based on multiplying reported frequency and duration, but they estimated duration based on categorical data – this is not appropriate unless there is validation information for this?

**Thank you for your relevant comment.** **The physical activity variable was coded to fit the units of current health recommendations, which is minutes per week. This process, which entailed multiplying the numerical values assigned to the answers to the questions regarding frequency and duration to obtain minutes per week has yet to be validated. The potential implications of this procedure have been now added in limitations in our discussion section (see line 345-349).**

**Furthermore, while exploring a dose-response relationship between education and physical activity would have been of great interest, this was outside the scope of our study. The study focuses explicitly on cut-off points for each variable as stated in current health recommendations, which we have now pointed out more clearly.**

• The decision to measure SES by education alone was confusing. The authors reasoned that this would be a good measure because it is stable, yet their study was longitudinal, so if their predictor is stable, what is the benefit of a longitudinal analyses, as this would make it less likely to observe changes in health behaviours as a result of changes in SES over time (because education does not change like occupation and income, which could affect the ability to ‘afford’ engaging in good health behaviours). The authors provided no hypotheses - is this what they expected to observe? This is indeed what they found, but this seems entirely predictable based on previous work and the nature/stability of this variable. What would have been more interesting is to examine interactions between education (stable variable) and occupation or income (unstable variable) which might help tease apart how these variables contribute to health behaviours. For example, education might be important for healthy eating, except when one loses their job and income – where buying healthy foods might be more difficult. Similarly, education might be associated with higher alcohol consumption (because drinking can be expensive and it is often seen as status symbol)- yet this association might decrease in those who lose their job or income.

**Thank you for your relevant comment. After careful consideration, we have now replaced the term “socioeconomic status” and left it as education level, to convey that the primary focus of the paper is investigating the association between education level and the patterns in both BMI and the other three health behavior variables. Furthermore, we have expanded the discussion section (lines 367-379) to thoroughly address both the positive and negative aspects of using education as a time-invariant variable, as you have rightly pointed out. In addition, it is important to acknowledge that in our study, most respondents had completed their education by the initial measurement point. On the other hand, salary is subject to more fluctuations over time, which adds complexity to the analyses involving this variable. Additionally, it should be noted that health behaviors can also impact income changes, and we have recognized this aspect as well. Moreover, Norway exhibits relatively small salary differences compared to other countries (1) making it a less influential factor in explaining changes in compliance with health-related guidelines. It is worth mentioning that income data in the Tromsø Study waves remain self-reported and have not undergone validation. Consequently, education, which has been validated (2), remains the optimal choice within the context of our study.**

**We fully agree that all studies need to have a clear aim. We believe that our aim is relatively clear, and that we have fulfilled that aim with the present work. Since our work is, as noted, more descriptive in its form, we chose not to formulate a formal hypothesis.**

• Table 1 was confusing and hard to interpret because of the high number of independent groups that were presented across each health behaviour (and BMI is not a health behaviour). It would be more helpful and informative to classify each behaviour as getting better, worse or staying the same over time as a function of sex.

**Thanks for your comment. The outcome of the MCA does precisely what the reviewer suggests, it presents the changes in the variables related to health behavior according to improvement, deteriorating or continuality, and was included separately for sex and age group. The reviewer mentioned that it could not be appreciated due to the low quality of the images. We apologize for that. However, the images were submitted according to the journal’s format requirement.**

• I was not able to read the figures because the resolution was not high enough. The text summarizing the results of the MCA analysis was also confusing – possibly because of the multiple analyses and largely descriptive nature of the findings.

**All the images were submitted according to the journal’s format requirements. If figures do not have the desired resolution, we believe that must be due to a conversion made by the journal when converting into PDF. We encourage the reviewers to access the figures through the links in the PDF or by asking the journal editorial team.**

• The discussion did not explain any of the mechanisms behind the observed associations, which really diminishes the contribution of the manuscript. The text on page 21, line 281 to 305, seems to go in circles and say no more than education is tied to health behaviours and this is generally maintained over time. How this is association may be explained by interactions with other risk factors was not discussed.

**Thank you for your comment. We have now added potential explanations behind the observed associations in our discussion section to provide useful insights to the reader.**

• As the authors acknowledged, there is a high risk of selection bias and self-report bias, which undermines confidence in the data.

**Thank you for pointing this out. The high risk of selection and self-report bias is discussed in lines 321-341.**

**References:**

**1.	Kinge JM, Modalsli JH, Øverland S, Gjessing HK, Tollånes MC, Knudsen AK, et al. Association of Household Income With Life Expectancy and Cause-Specific Mortality in Norway, 2005-2015. Jama. 2019;321(19):1916-25.**

**2.	Vo CQ, Samuelsen P-J, Sommerseth HL, Wisløff T, Wilsgaard T, Eggen AE. Validity of self-reported educational level in the Tromsø Study. Scandinavian journal of public health.0(0):14034948221088004.**
